# Replication of Influenza D Viruses of Bovine and Swine Origin in Ovine Respiratory Explants and Their Attachment to the Respiratory Tract of Bovine, Sheep, Goat, Horse, and Swine

**DOI:** 10.3389/fmicb.2020.01136

**Published:** 2020-05-25

**Authors:** Eva Mazzetto, Alessio Bortolami, Alice Fusaro, Elisa Mazzacan, Silvia Maniero, Marta Vascellari, Maria Serena Beato, Eliana Schiavon, Chiara Chiapponi, Calogero Terregino, Isabella Monne, Francesco Bonfante

**Affiliations:** ^1^Istituto Zooprofilattico Sperimentale delle Venezie (IZSVe), Legnaro, Italy; ^2^Istituto Zooprofilattico Sperimentale della Lombardia e dell’Emilia Romagna “Bruno Ubertini” (IZSLER), Brescia, Italy

**Keywords:** influenza D virus, ovine, explant, attachment, phenotype

## Abstract

Bovine is considered the main reservoir of influenza D virus (IDV), however, low levels of seropositivity in other farmed species suggest a wide range of potential hosts. Nevertheless, it is not clear whether this scenario is the result of rare spillover events upon contact with bovines, or a lack of adaptation of IDV to these hosts. Among these species, sheep represents a crucial component of the rural economy in many developing countries, but little is known about its role in the ecology of the disease. To evaluate the susceptibility of sheep to IDV viruses of different origin, we used ovine respiratory tissues as an *ex vivo* model and investigated the infective phenotype of two IDV strains isolated from either bovine (IDV-BOV) or swine (IDV-SW). For translatability purposes, we included a parainfluenza type 3 virus, as positive control, given its known respiratory tropism in sheep. We performed a timed evaluation of the viral infectivity, cell tropism and the associated histopathology, by means of tissue culture infectious dose assays on supernatants and histological/immunohistochemical analyses on explanted tissues, respectively. To further investigate differences in the phenotype of these two strains and to identify the potential targets of replication in the most commonly land-based farmed mammalian species, we carried out virus binding assays on histological sections of the respiratory tract of bovine, caprine, ovine, horse and swine. Our results demonstrated that IDV successfully replicates in nasal, tracheal and lung ovine tissues, suggesting a moderate susceptibility of this species to IDV infection. Interestingly, despite the high genetic identity of these strains, IDV- BOV consistently replicated to higher titers than IDV-SW in all respiratory tracts, suggesting IDV viruses might display considerable levels of variability in their phenotype when crossing the species barrier. Virus binding assays confirmed a superior affinity of the IDV viruses for the bovine upper respiratory tract, and a preference for the pharyngeal epithelium of small ruminants, indicating possible targets to improve the sensitivity of virological sampling for diagnostic and post-mortem purposes. Further pathogenesis and cross-species transmission studies will be necessary to elucidate the ecology of IDV and eventually allow the design of cost-effective surveillance strategies.

## Introduction

Influenza D virus (IDV) is a newly described influenza type of the Orthomyxoviridae family, distantly related to Influenza C virus (Hause et al., 2014). IDV was first isolated from a swine exhibiting influenza-like symptoms in Oklahoma, in 2011 (Hause et al., 2013) but serological evidence has indicated circulation of the virus among cattle in Nebraska, since 2003 (Luo et al., 2017). Although the susceptibility of pigs to IDV has been demonstrated by detection of the virus during respiratory disease outbreaks and confirmed by means of experimental *in vitro* and *in vivo* studies: a growing body of data generated by active and passive surveillance activities, indicates swine as a minor host for IDV (Foni et al., 2017; Ferguson et al., 2018; Snoeck et al., 2018; Sreenivasan et al., 2019). On the other hand, the frequent isolation of IDV from cattle together with a high seroprevalence in bovine herds, indicate this species as the main reservoirs of IDV (Oliva et al., 2019). In experimental settings, bovine directly infected with IDV exhibited mild respiratory signs and minimal epithelial damage, while in the field IDV has been consistently associated with overt respiratory distress (Ferguson et al., 2016; Hause et al., 2017; Salem et al., 2019). Metagenomics studies demonstrated that IDV might be associated with the bovine respiratory disease complex (BRDC), a multifactorial respiratory infection severely affecting the economy of meat production (Mitra et al., 2016). The ethiological role of IDV in BRDC poses a serious challenge to the beef industry, as this virus is endemic in North America and widely circulates in Asia, Europe, and Africa and no commercial vaccine is currently available (Ducatez et al., 2015; Chiapponi et al., 2016; Horimoto et al., 2016; Salem et al., 2017; Zhai et al., 2017; Snoeck et al., 2018). Based on the Hemagglutinin-esterase (HEF) gene, at least four main genetic and antigenic clusters have been identified, namely D/OK, D/660, D/Japan, and D/Yama2019 (Murakami et al., 2020). Besides cattle and pigs, small ruminants, horses, camelids and feral swine resulted to be serologically positive for IDV, suggesting a broad host-range for this virus (Quast et al., 2015; Nedland et al., 2017; Salem et al., 2017; Ferguson et al., 2018; Murakami et al., 2019; Oliva et al., 2019). Despite the abundant serological data available, little is known regarding the pathogenic potential of IDV in land-based mammalian farmed species. Among these species, small ruminants are considered a low-risk investment for their short reproduction cycle and versatility in a changing environment, as they can live in arid, as well as in semi-tropical conditions and are able to feed on a wide variety of plants, converting this energy into meat, milk, fibers, manure, and skins (Akinmoladun et al., 2019). Of the world’s 1.6 billion sheep, 65% of them are located in developing countries, where they are farmed in small-scale holdings often implementing multi-species grazing systems, together with goats and cattle (Organizacion de las Naciones Unidas Para la Alimentacion y la Agricultura, 1986; Hardy and Tainton, 1995). Given their geographical distribution, farming system and the indication of varying degrees of IDV seroprevalence, we believe more research should be done to evaluate the ecological role of sheep in the IDV circulation. Besides, sampling for IDV in small ruminants in Italy is often limited to swabbing of the nostrils *in vivo* and to collection of lung specimens during post-mortem examination in case of respiratory illness, but these practices are not based on experimental evidence and might suffer from a suboptimal sensitivity. To expand the body of knowledge in these areas and better understand whether host-origin could play a role in the ecology of the disease, we performed both a genotype and phenotype characterization of Italian IDV strains (IDVs) isolated from bovine and swine during surveillance activities. In particular, we evaluated the replicative phenotype of these isolates in ovine respiratory explants of nose, trachea and lung. Moreover, we compared the potential receptivity of bovine, pig, horse, sheep and goat respiratory tissues to these viruses, studying *in vitro* the profile of viral attachment, aiming to both identify the most receptive host and tissue within each host.

## Materials and Methods

### Viruses

In this study, two IDV strains, a parainfluenza type 3 virus (PI3V) and an avian influenza virus (AIV) were used. The IDVs were isolated from either a bovine or a pig nasal swabs collected during surveillance activities in Italy. The influenza D strain D/bovine/Italy/17VIR1851/2016 (IDV-BOV) was isolated on Human Rectal Tumor 18G (HRT-18) cells and screened for the presence of the main respiratory viruses causing disease in cattle. The swine isolate of influenza D virus, D/swine/Italy/18VIR6833/2015 (IDV-SW) was kindly provided by Istituto Zooprofilattico Sperimentale della Lombardia e dell’Emilia Romagna (IZLER). The IDV-BOV and IDV-SW strains were selected based on previously conducted phylogenetic analysis (data not shown) that described these viruses as representatives of the most common lineage circulating in both swine and bovine populations in Europe (Foni et al., 2017). Selection of viruses isolated from the same anatomical region was intentional, to minimize the possibility that phenotypic and genotypic differences could depend on adaptation to the tissue of origin rather than the host. The IDVs were propagated in Madin-Darby Canine Kidney cells (MDCK) cells in minimum essential medium eagle (MEM) supplemented with 0.1% (1 μg/ml) TPCK Trypsin (Sigma-Aldrich, United States) and 1% penicillin and streptomycin (Sigma-Aldrich, United States). The H10N7 A/mallard/Italy/05VIR4290-6/2005 AIV (H10) was isolated from a dead wild mallard during Italian surveillance activities (Bonfante et al., 2014) and was chosen as a control virus for low replication, assuming that wild bird isolates of avian influenza have only sporadically been associated with seroconversion in small ruminants (Reperant et al., 2009). A stock of the H10 virus was produced by inoculating 9-to-11-day-old embryonated specific pathogen free (SPF) hen’s eggs. IDVs and H10 viruses were titrated by the plaque forming unit (PFU) assay on MDCK cells according to Matrosovich et al. (2006). We chose a strain of PI3V, isolated from a bovine diagnostic sample, as positive control virus for the infection of ovine respiratory explants. A stock of PI3V was produced by replicating the virus on Madin-Darby Bovine Kidney cells (MDBK) in MEM supplemented with 5% FCS. After 2 days the cellular supernatant was harvested and titrated in MDBK cells by standard tissue culture infectious dose 50% (TCID_50_) assay using the Reed and Muench formula (Reed and Muench, 1938), the titer was converted in PFU applying the formula PFU = 0.69 × 1 TCID_50_ based on the Poisson distribution.

### Sequencing, Phylogenetic, and Molecular Analysis

We sequenced the genome of 22 IDV positive clinical samples of lung or tracheal swab collected between 2015 and 2018 from bovine, in Northern Italy. RNA from clinical samples was extracted using the QIAamp Viral RNA Mini Kit (QIAGEN, Milan, Italy) according to the manufacturer’s instructions. Complete IDVs genome were amplified with the SuperScript III One-Step RT-PCR system with Platinum Taq High Fidelity (Invitrogen, Carlsbad, CA, United States) using one pair of primers complementary to the non-coding regions of IDV as previously described by Hause et al. (2014). Sequencing libraries were obtained using Nextera XT DNA sample preparation kit and processed on an Illumina MiSeq instrument with MiSeq reagent kit V2 (2 × 150 PE mode) or V3 (2 × 300 bp PE mode) (Illumina, San Diego, CA, United States).

Illumina reads quality was assessed using FastQC v0.11.2; raw data were filtered by removing: (i) reads with more than 10% of undetermined (“N”) bases; (ii) reads with more than 100 bases with Q score below 7; (iii) duplicated paired-end reads. Remaining reads were clipped from Illumina Nextera XT adaptors using scythe v0.991^1^ and trimmed with sickle v1.33^2^. Reads shorter than 80 bases or unpaired after previous filters were discarded. High quality reads were aligned against a reference genome using BWA v0.7.12 (Li and Durbin, 2010). Alignments were processed with Picard-tools v2.1.0^3^ and GATK v3.5 (McKenna et al., 2010; Depristo et al., 2011; van der Auwera et al., 2013) to correct potential errors, realign reads around indels and recalibrate base quality. Single Nucleotide Polymorphisms (SNPs) were called using LoFreq v2.1.2 (Wilm et al., 2012), and the outputs were used to generate consensus sequences.

Sequences were submitted to GenBank under accession numbers MK965257 to MK965388. All IDV sequences available from GenBank on the 14th February 2019 were downloaded and aligned with sequences generated in this study using MAFFT v. 7 (Katoh and Standley, 2013). We inferred maximum likelihood (ML) phylogenetic trees for each gene segment using IQ-Tree v1.6.9 (Nguyen et al., 2015) and the best-fit model of nucleotide substitution determined with ModelFinder (Kalyaanamoorthy et al., 2017). Ten thousand ultrafast bootstrap replicates were performed to assess the robustness of individual nodes of the phylogeny. Phylogenetic trees were visualized with the program FigTree v1.4.2^4^. To assess whether IDV showed a significant clustering by host (bovine/swine) we used the BaTS program (Parker et al., 2008) to estimate values of the association index (AI), parsimony score (PS), and maximum monophyletic clade (MC) statistics of phylogeny-trait association with the traits (hosts) for each gene segment. This method compares a posterior distribution of trees to a null distribution of 1,000 trait-randomized trees. To obtain the posterior sample of trees we inferred non-clock Bayesian trees for each of the seven gene datasets using MrBayes v3.2.6 and the GTR + Γ4 nucleotide substitution model.

### Selection Pressure Analysis

Site-specific selection pressure for all segments of IDV was measured as ratio of non-synonymous (dN) to synonymous (dS) nucleotide substitution per site. dN/dS ratios were estimated using the mixed effects model of evolution (MEME), which allows to reliably detect sites subjected to both pervasive and episodic positive selection (Murrell et al., 2012), and the fixed-effect likelihood (FEL) (Kosakovsky Pond and Frost, 2005) methods available at Datamonkey online version of the Hy-Phy package (Kosakovsky Pond et al., 2005; Weaver et al., 2018), as test designed to detect pervasive positive selection.

### Animals for Explant Collection

Three clinical healthy 3-to-5-month-old lambs from a herd with high biosecurity standards were selected. The animals were tested for the presence of IDV and IAV, analyzing nasal swabs by rRT-PCR (Spackman et al., 2003; Hause et al., 2014), and their sera were screened for the presence of antibodies against IDV, IAV and PI3V, using, respectively, a hemagglutination inhibition test (HI) for the influenza viruses and SVANOVIR^®^ PIV3-Ab ELISA test for the PI3V (SVANOVA, Milan, Italy). For 3 days before the explant procedure, the animals were treated with enrofloxacin (Baytril 10%, Bayer S.p.a., Milan, Italy) at a dosing regimen of 5 mg/kg body weight, to reduce bacterial load at the level of the respiratory tract. After the treatment, the lambs were transported from the farm to the facilities of Istituto Zooprofilattico Sperimentale delle Venezie (IZSVe) and euthanasia was performed by exsanguination, under anesthesia. Animal experimental procedures were conducted in strict accordance with the Decree of the Ministry of Health n. 26 of March 04, 2014 on the protection of animals used for scientific purposes, implementing Directive 2010/63/EU. Authorization N°8B654. NDJA was obtained from the Italian Ministry of Health.

### Culture of Respiratory Explants

To cover the ovine respiratory apparatus, sections of nose, trachea and lung were selected to be cultivated *ex vivo*. Explants were isolated and prepared according to the van Poucke et al. (2010) protocol. Briefly, nasal mucosa was stripped from the medial turbinate and cut in squares of 50 mm^2^ and cultivated in an air-liquid interface, in 6-well plates at 37°C with 5% CO_2_. Each well contained 3.2 ml of medium [50% RPMI Medium 1640 (1X) + L-glutamine (Gibco, Life Technology, Monza, Italy) 50% DMEM GlutaMAX^TM^- Pyruvate (Gibco, Life Technology, Monza, Italy), penicillin 100 U/ml (Gibco, Life Technology, Monza, Italy), streptomycin 100 μg/ml (Gibco, Life Technology, Monza, Italy), gentamycin 0.1 mg/ml (Gibco, Life Technology, Monza, Italy)]. After the cartilage and adventitia were removed, the trachea was processed and cultivated as done for the nasal mucosa, replacing gentamycin with 2.5 μg/ml of Amphotericin B (Gibco, Life Technology, Monza, Italy) in the medium and using DMEM GlutaMAX^TM^- + Pyruvate (Gibco, Life Technology, Monza, Italy). Lung explants were obtained from the right apical lobe. The lung was perfused with a 1% (w/v) solution of a low-melting temperature agarose (Sigma-Aldrich, United States) and let solidify at 4°C, for 20 min (mins). The solidified lung was then cut into sections of 1–2 cm^3^ that were transferred into a 20 ml syringe filled with a 4% (w/v) low-melting temperature agarose. A 20 min incubation at 4°C allowed the complete embedding of the solidified lung in the syringe. The embedded lung tissue was cut into slices of 1 mm thick, using a cryotome blade. These slices were trimmed until they had a surface of 25 mm^2^ and incubated overnight in 24-well plates containing 1.4 ml of medium [DMEM GlutaMAX^TM^- Pyruvate (Gibco, Life Technology, Monza, Italy), penicillin 100 U/ml (Gibco, Life Technology, Monza, Italy), streptomycin 100 μg/ml (Gibco, Life Technology, Monza, Italy), gentamycin 0.1 mg/ml (Gibco, Life Technology, Monza, Italy), human insulin 2.5 μg/ml (Sigma-Aldrich, United States), retinyl acetate 0.5 μg/ml (Sigma-Aldrich, United States), hydrocortison 0.5 μg/ml (Sigma-Aldrich, United States) at 37°C and 5% CO_2_]. To evaluate the organ culture viability, nasal and tracheal explants were checked daily for ciliar beating by light microscopy.

### Explant Infection and Virus Titration of Supernatants

At least six biological replicates were conducted for each explant tissue and for each virus type. Infection was performed as described in van Poucke et al. (2010) dosing explants with 10^6^ PFU in 600 μl of culturing medium. We collected 300 μl of supernatant at 1, 24, 48, and 72 h post inoculation and replaced it with fresh medium. To assess virus yields, 10-fold dilutions of the supernatants collected from explants infected with IDVs and H10 were inoculated onto MDCK cells grown in 96-well plates. The plates were incubated for 3 days, at 37°C with 5% CO_2_ and the supernatants checked for hemagglutinating activity, at 72 h post-infection (p.i.). The titration of supernatants collected from explants infected with PI3V was performed with the same procedure, inoculating onto MDBK and evaluating for the cytopathic effect, at 72 h p.i. For all of the samples the titer was calculated by the method of Reed and Muench (1938) and expressed as TCID_50_/ml.

### Histology Evaluation and Immunohistochemistry

Non-infected negative control explants were collected at 0, 24, 48, and 72 h post collection and fixed for 48 h in 10% (v/v) formalin and subsequently stained as a 4 μm histologic section with hematoxylin and eosin. All infected explants were collected at 72 h p.i., fixed in formalin 10% for at least 48 h to subsequently perform histological and immunohistochemical examinations. The immunohistochemistry (IHC) was carried out on 3-μm sections by the BenchMark ULTRA automated immunostainer (Ventana Medical Systems, Tucson, AZ, United States). The sections were dewaxed, at 72°C for 8 min. For tissues infected with PI3V, antigen retrieval was performed using the commercial pre-diluted ULTRA cell conditioning (CC2) solution (Ventana Medical Systems, Tucson, AZ, United States) at pH 6 at 95°C for 36 min, while for tissues infected with either IAV or IDVs, Protease 2 (Ventana Medical Systems, Tucson, AZ, United States) was used at 36°C for 12 min. Depending on the virus used for the infection, the slides were then incubated with one of the following antibodies: a polyclonal rabbit anti-IDV antibody (in-house produced) applied at 1:1000 dilution, a mouse monoclonal anti-IAV (Clone 1331, Meridian, Memphis, TN, United States) applied at 1:1500 dilution, a mouse monoclonal anti-PI3V (cod. BIO 290, Bio-X Diagnostics), used at 1:20 dilution. The incubation of each antibody lasted for 80 min at RT. The staining was revealed with an indirect biotin-free UltraView universal DAB detection kit (code 052 697 806 001, Ventana Medical Systems, Tucson, AZ, United States). All of the tested samples were counterstained with hematoxylin.

### Virus Purification, Inactivation, and Labeling for Virus Binding Assays

The concentration and purification of the IDVs was carried out modifying a published protocol (Hutchinson et al., 2014). Briefly, after replication of the viruses on MDCK, the cellular supernatant was collected and centrifuged at 3,000 *g* for 30 min, at 4°C. The clarified supernatant was centrifuged at 112.000 *g* in SW28 (Beckman Coulter Optima L-100 Ultracentrifuge) for 2 h, at 4°C, and subsequently, the pellet was suspended in phosphate buffered saline (PBS) and loaded onto a 30–60% (w/v) sucrose gradient and centrifuged at 209,000 *g*, in SW21 for 2.5 h, at 4°C. The purified virus was collected and the sucrose removed by centrifugation at 112,000 *g* in SW28 for 2 h, at 4°C.

Inactivation and labeling of the purified influenza D virus were done according to van Riel et al. (2007). Inactivation was carried out by dialysis against 0.1% formalin for 72 h. After inactivation, the virus was dialyzed against formalin in PBS. The inactivated virus was labeled through a 1 h incubation at room temperature (RT) with a 1:1 (v/v) 0.5 mol/L bicarbonate buffer solution (pH 9.5) containing 0.1 mg/ml of FITC (Sigma-Aldrich, United States). The virus was dialyzed overnight in PBS to release unbound FITC, using Pur-A-Lyzer tubes (Sigma-Aldrich, United States).

### Virus Binding Assays

To compare the binding preference to sialic acids (Sia) of IDV-BOV and IDV-SW, we performed a HA using red blood cells (RBCs) collected from different animal species. Chicken, turkey, and horse RBCs were used to evaluate HA of IDV, while bovine RBCs were used as negative control (Sreenivasan et al., 2015; Eckard, 2016; Luo et al., 2017; Salem et al., 2019). The HA was performed according to OIE guidelines for avian influenza viruses (OIE, 2015), incubating virus serial dilutions with either 1% chicken or 1% turkey RBCs for 30 min, while an incubation of 1 h was carried out for both 1% horse and 1% bovine RBCs. HA titers were determined as the highest dilution to fully agglutinate the RBCs. HA titers obtained using different RBCs were normalized and expressed as percentage value in relation to the titer obtained with turkey RBCs and represented as a percentage. The assays were performed as technical duplicates for three times.

To study the binding profile of IDV-BOV and IDV-SW and infer the potential tissue tropism and host preference, we compared the attachment ability of IDVs along the respiratory tract of bovine, pig, horse, sheep and goat, relying on the virus-histochemistry technique (VHC). For each of these species we collected tissue sections of the nasal turbinates, pharynx, trachea, and lung. Tissues were sampled from healthy animals at the slaughterhouse and immediately fixed in 10% formalin. Each tissue sample was subsequently paraffin embedded and evaluated in hematoxylin and eosin for the absence of histological lesions. The virus histochemistry technique was performed on the collected tissues according to the protocol developed by van Riel et al. (2007). Shortly, 3-μm sections were deparaffinized and hydrated with graded alcohols. Incubation of 16 hemagglutinin units (HAU) of the labeled virus was carried out overnight, at 4°C. To visualize the bound virus, slides were incubated with a monoclonal antibody targeting the FITC molecule conjugated with horseradish peroxidase (PerkinElmer, Waltham, MA, United States). The signal was amplified by a tyramide-biotin signal amplification system (PerkinElmer, Waltham, MA, United States). Peroxidase was revealed with 3-amino-9-ethyl-carbazole (AEC) (Sigma-Aldrich, United States). Tissues were counterstained with hematoxylin and embedded in glycerol.

### Statistical Analysis

Data were analyzed with GraphPad Prism5 software (GraphPad Software Inc., San Diego, CA, United States). In the infection of explants, significant differences among viruses yields at each p.i. time in nose, trachea and lung were evaluated by analysis of variances using one-way ANOVA followed by Tukey’s *post hoc* test. If homoscedasticity of the variables was not met as assessed by Barlett’s test, the data were log-transformed prior to ANOVA. In HA, a non-parametric *t*-test (Mann–Whitney test) was applied to determine a significant difference in hemagglutinating activity between IDV-BOV and IDV-SW. Differences were considered to be statistically significant at *p* < 0.05.

## Results

### Phylogenetic and Molecular Analysis

**FIGURE 1:**
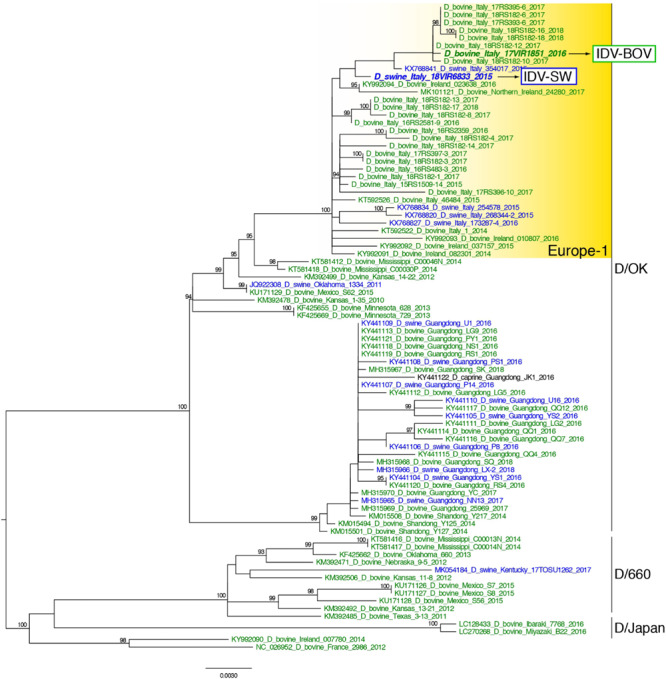
Maximum likelihood phylogenetic tree of the HEF gene segment of influenza D viruses. Viruses are colored according to the host species: blue for swine, green for bovine. The two strains characterized in this study are in bold. Europe-1 cluster, including all the Italian viruses, is highlighted in yellow. The numbers at the nodes represent ultrafast bootstrap values (>90%).

Topology of the maximum likelihood phylogenetic trees of the seven gene segments showed that all of the Italian viruses collected between 2014 and 2018 fell within lineage D/OK and clustered with viruses from Ireland (group Europe-1) (Figure 1 and Supplementary Figures S1–S6). Despite the limited number of sequences from swine, no clustering of influenza D viruses by host was observed. This data was supported by the phylogeny-trait association test, which revealed for all the genes no statistically significant structuring by host (*P* > 0.04 for bovine and *P* > 0.06 for swine, Supplementary Table S1). IDV-BOV and IDV-SW clustered separately within the Europe-1 group, showing a mean genetic identity of 99.53% with a total of 16 amino acid substitutions distributed across the HEF (positions 17, 276, 289, 385, and 518), NS1 (positions 112 and 140), NS2 (positions 12 and 71), NP (position 383 and 411), P42 (52, 296, 304, and 366), and PB1 (position 72) genes (Table 1).

**TABLE 1:** Amino acid differences between IDV-BOV and IDV-SW.

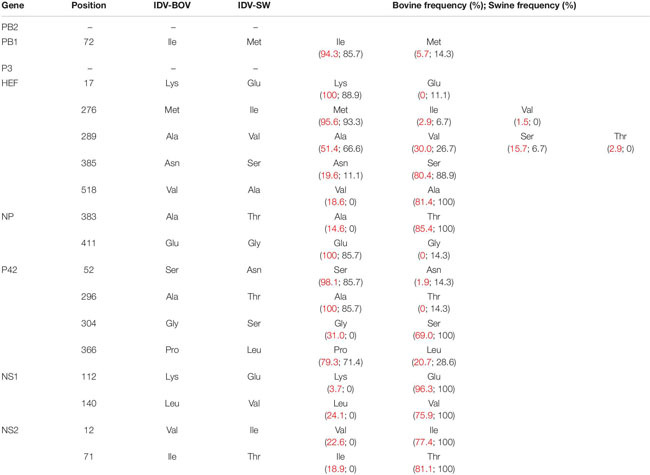

*Identification of different amino acids between IDV-BOV and IDV-SW IDV strains. Percentages in brackets indicate the relative frequency of amino acidic substitutions in the available sequences of viruses of either bovine (in red) or swine (in black) origin.*

In the molecular analyses, numbering of amino acids started from the first methionine. We observed that some of the amino acid substitutions were unique of the viruses under study, while others were observed only in viruses collected from one host species. In particular, three substitutions appear to be unique of viruses from swine, namely HEF-E17, NP-G411, and P42-T296, while seven have been identified exclusively in bovine viruses (HEF-518V, NP-383A, P42-304G, NS1-112K, NS1-140L, NS2-12V, and NS2-71I). Interestingly, we found 12 substitutions showing evidence of putative positive selection using the MEME and/or FEL methods (*P* < 0.1, Supplementary Table S2). In particular, at one of these positions (289), which was consistently identified by both methods (*P* < 0.1, Supplementary Table S2), the IDV-BOV and IDV-SW viruses carry an alanine and a valine, respectively, two variants that are equally distributed in the two species according to the available sequences in GenBank (Table 1).

### Replication of IDVs in Ovine Respiratory Explants

**FIGURE 2:**
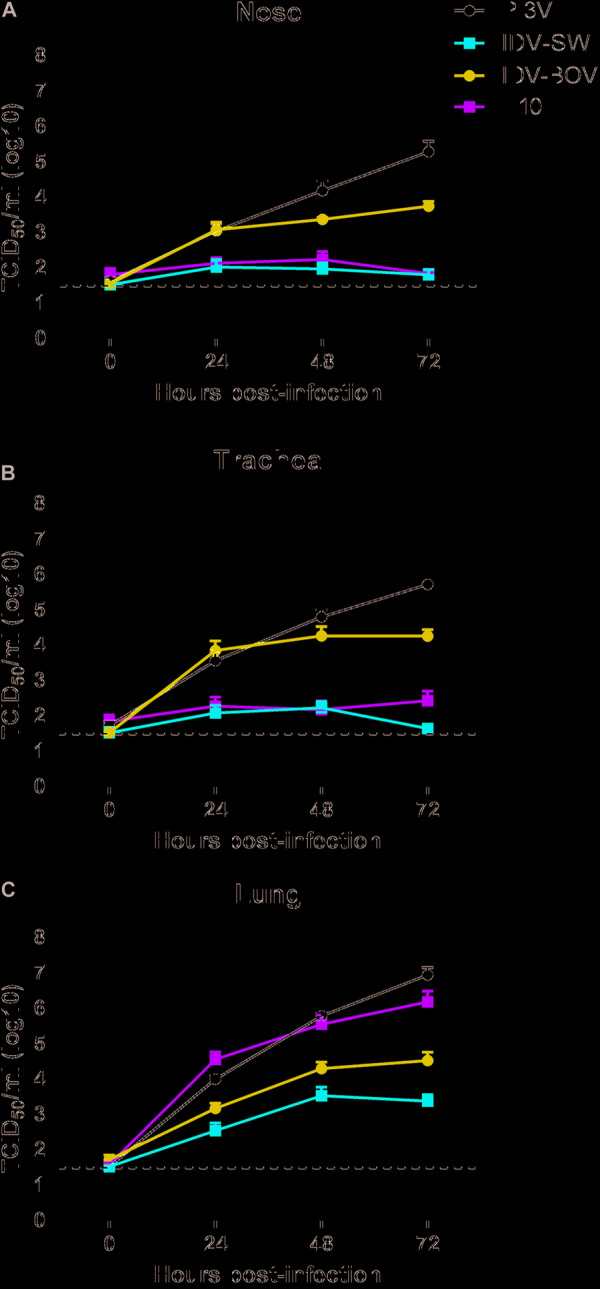
Viral growth curves in explants of ovine respiratory tissues. Ovine nasal **(A)**, tracheal **(B)**, and lung **(C)** explants from three sheep were inoculated with 10^6^ FFU of IDV isolated from bovine (IDV-BOV) or swine (IDV-SW), parainfluenza 3 virus (PI3V), avian influenza H10N7 (H10). Aliquots of explant supernatants were collected at 0, 24, 48, and 72 h post infection and analyzed by tissue culture assay. Data represent the means ± the standard error of mean (SEM) from six explants per experiment under each condition and they are expressed as log 10 of tissue culture infection dose 50 per ml (TCID_50_/ml). The dashed lines indicate the limit of detection of tissue culture assay.

All animals used for the collection of explants resulted negative for IDV, IAV, and PI3V by RRT-PCR and were seronegative for the same viruses. Monitoring of ciliary motility by macroscopic observation of nasal and tracheal explants at the light microscope indicated functional integrity up to 72 h post culturing. Moreover, eosin and hematoxylin staining of non-infected explants showed a well-conserved histological architecture for the same period of time (data not shown). PI3V recorded the highest titers in all tracts by 72 h p.i., while IDVs and H10 although replicating in all of the tissues, showed different degrees of fitness (Figure 2). In the nasal and tracheal mucosa, IDV-BOV recorded mean values of TCID_50_ 1–3 log10 higher than IDV-SW for which statistical significance was proved, at 24–72 h p.i. In these tissues, IDV-SW replicated to levels in the range of one log10 above the limit of detection of the TCID_50_ assay and comparable to the ones of H10, as differences between the two viruses were non-significant. In the lung system, PI3V and H10 recorded the highest values and PI3V resulted to be significantly higher than titers observed for both IDVs, at 24–72 h p.i. IDV-BOV replicated approximately one log10 higher than IDV-SW at all time-points and differences were statistically supported.

### Cell Tropism of IDV in Ovine Respiratory Explants

**FIGURE 3:**
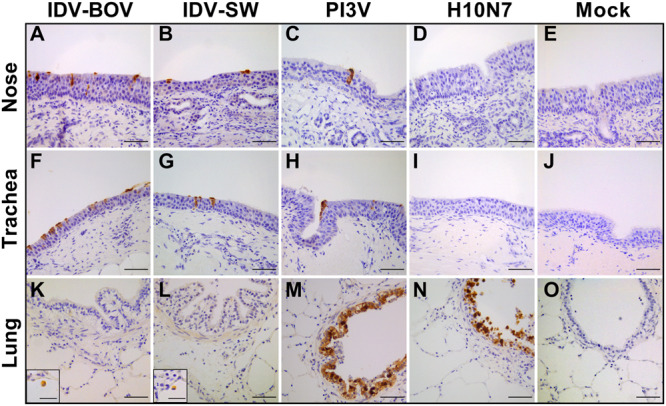
Immunohistochemical analysis of infected explants. Each image represents nasal **(A–E)**, tracheal **(F–L)**, or lung **(K–O)** ovine explants infected with IDV isolated from bovine (IDV-BOV) or swine (IDV-SW), parainfluenza 3 virus (PI3V), avian influenza H10N7 (H10) or mock infected and collected at 72 h p.i. In the images **(M,N)**, the insertion shows a magnification of a positive alveolar macrophage in lung explant, respectively, infected by IDV-BOV and IDV-SW. Scale bar = 50 μm.

Immunohistochemistry results are presented in Figure 3. PI3V was detected in all types of explants. In the nose and trachea, staining was limited to the nuclei and cytoplasm of ciliated cells at the luminal side of the respiratory epithelium, while in the lung, positivity localized in the ciliated bronchiolar and non-ciliated Clara cells lining the lumen of the bronchioles. PI3V-positive bronchioles showed signs of degeneration and necrosis (data not shown). For both IDV strains, we identified IHC-positive cells almost exclusively in the explants of the upper respiratory tract. In both the nose and the trachea, IDVs replicated in the ciliated cells of the epithelium, while in the lung, positivity was rare and limited to the alveolar macrophages. IDV-BOV and IDV-SW did not induce evident pathological changes, as suggested by the identical histological appearance between infected and control explants. H10 replicated in the ciliated and non-ciliated cells lining the bronchiolar lumen causing extensive degeneration and necrosis.

### Attachment of IDVs to the Respiratory Apparatus of Bovine, Sheep, Goat, Horse, and Pig

**FIGURE 4:**
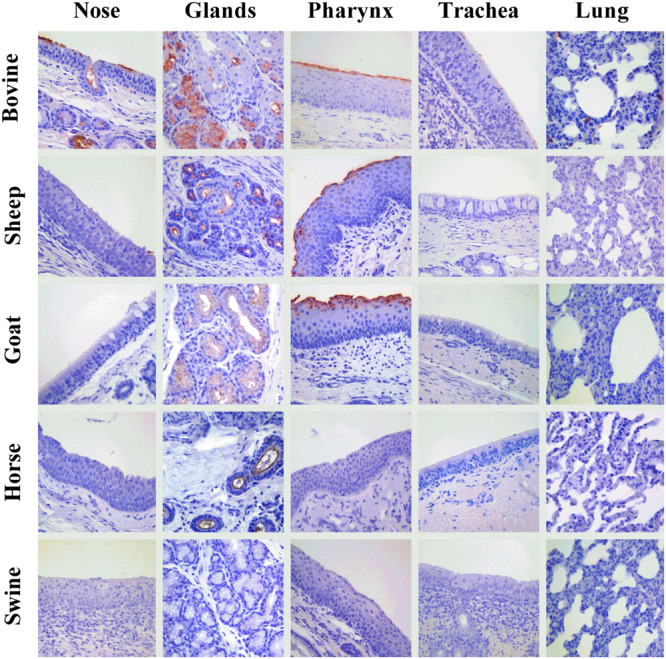
Attachment of IDV to respiratory tissues of bovine, sheep, goat, horse, and pig. Influenza D virus (IDV) attachment (in red) to respiratory tissues of main agriculture species resulted seropositive for IDV in the field. Each column represents a section of the respiratory tract: from the left, the nasal turbinate (nose), submucosal glands of upper respiratory tract (glands), pharynx, trachea, and lung. Each row indicates the animal species: from the top, bovine, sheep, goat, horse, and swine. The image acquisition was performed at 400× magnification.

An overview of the attachment of IDVs to each tissue and host is summarized in Table 2 and Figure 4. Given the normal histological appearance of the epithelia, tissues were deemed suitable for the VHC assay. IDV-BOV and IDV-SW showed identical binding profiles on the tested tissues. In bovine, IDVs mainly attached to the surface of the nasal turbinates, in particular at the level of the apical side of ciliated cells where cilia strongly stained. In the same region, positivity was abundant in the lumen of secretory tubes and ducts of submucosal glands, where precipitate was clearly visible both inside the cytoplasm of mucous cells and at their apical side. In the pharynx, IDVs robustly attached to the luminal side of the pseudostratified epithelium and it was visible as a red uniform precipitate. Virus attachment to the tracheal epithelium was sparse but evenly distributed with a dot-like appearance at the level of the cell membrane on the apical side of ciliated epithelial cells, while cilia in this case rarely stained. In bovine lung, IDVs binding was limited to few areas of the tissue section. In those areas, virus specifically attached to alveolar cuboid cells identified as type II pneumocytes. In the nasal turbinate of sheep, IDVs rarely attached to ciliated cells of the respiratory epithelium, while virus binding was more abundant inside the cytoplasm of mucous cells and at the level of the lumen of submucosal glands. In the pharynx, IDVs attached to the surface of the pseudostratified epithelium and to mucous cells lining the lumen of submucosal glands. No attachment was observed either in the trachea or in the lung. In the goat, IDVs showed a pattern of viral binding similar to the one observed on ovine tissues with the exception of the nasal turbinate, where no attachment was detected. In the respiratory tissues of horse, staining was exclusively observed in the submucosal glands of the upper respiratory tract. IDVs did not attach to any respiratory tissues of swine. This result was further confirmed performing the assay on tissues collected from three animals of different age and geographical areas (data not shown).

**TABLE 2:** Score of IDV attachment in the respiratory tract of bovine, sheep, goat, horse, and pig.

	**Nasal turbinate**	**Submucosal glands**	**Pharynx**	**Trachea**	**Lung**
Bovine	+++	+++	+++	++	++
Sheep	+	+++	+++	−	−
Goat	−	+++	+++	−	−
Horse	−	++	−	−	−
Swine	−	−	−	−	−

*The mean abundance of cells to which virus attached was scored according to the methods published by van Riel et al. (2007) and it was applied as follow: − no attachment; + attachment to few cells, ++ attachment to a moderate number of cells; +++ attachment to many cells.*

### Hemagglutinating Activity of IDVs Isolates

**FIGURE 5:**
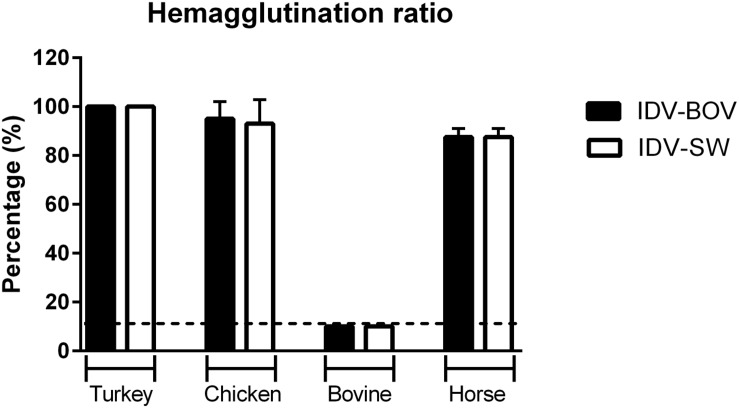
Hemagglutinating ability of IDVs isolates. Hemagglutinating ability of IDV isolated from bovine (IDV-BOV) or swine (IDV-SW), was expressed as percentages of hemagglutination (HA) titers of turkey, chicken, bovine, horse red blood cells (RBCs), and the respective HA of turkey RBCs titer. Each HA was performed in duplicate and the experiment was conducted three times. To determine a significant difference (*p-*value < 0.05) in HA between IDV-BOV and IDV-SW a Mann–Whitney non-parametric test was applied.

To compare the binding preference between IDV-BOV and IDV-SW, we performed HA using a panel of RBCs obtained from avian a mammalian species. IDV-BOV and IDV-SW showed the same hemagglutinating activity, irrespective of the type of RBCs, recording the highest HA titers with turkey RBCs, while no agglutination was observed with bovine RBCs (Figure 5).

## Discussion

Influenza D virus is considered a respiratory pathogen of bovine and there are evidences that it plays a role in the pathogenesis of BRDC (Collin et al., 2015). Although IDV has the ability to infect several mammalian species other than bovines, the resulting seroprevalence in these species is rather low compared to cattle (Snoeck et al., 2018; Oliva et al., 2019). It is not clear whether this scenario is the consequence of rare spillover events upon contacts with bovines, a lack of adaptation of this virus to other hosts, a variability in viral phenotypes, or simply the result of different farming systems and environmental conditions. Such a gap in knowledge clearly undermines the possibility of monitoring the circulation, evolution and spread of the virus in the different species.

In this study, we aimed at understanding whether IDV was able to replicate in sheep, as this species together with goat might play a crucial role in perpetuating the circulation of IDV in developing rural areas (Akinmoladun et al., 2019).

To test this hypothesis, we established an *ex vivo* model of sheep respiratory tract and investigated the infective phenotype of two Italian IDVs isolated from either bovine or swine hosts.

In our *ex vivo* model, IDVs proved to infect and actively replicate in the ovine nose, trachea and lung. Immunohistochemical data supported the active replication of both IDVs in nasal and tracheal tissues, showing a moderate to abundant positivity of the respiratory epithelial cells. Despite substantial replication in the lung, few alveolar macrophages were the only IHC-positive cells. This apparent discrepancy could be explained by either a high virus replication in relatively few macrophages, or replication in an overall high number of infected macrophages, due to the bigger mass of lung explants compared to nose and trachea. Although a direct comparison of replication between different explant types would not be accurate due to the technical impossibility of standardizing the multiplicity of infection, relative comparisons with a positive control virus are acceptable. In light of this, an overall evaluation of the replicative and immunohistochemical profiles of IDV and those of a common viral respiratory pathogen of sheep, brought us to infer that *in vivo*, IDV might act as a respiratory pathogen with a clear preference for ciliated epithelial cells of the upper respiratory tract, rather than bronchial and lung tissues. Since IDV replicated significantly less then PI3V and considering that PI3V is commonly associated with mild and often subclinical interstitial pneumonias in the field (Contreras-Luna et al., 2017), it is safe to assume that minimal clinical impact might be expected during IDV infections in sheep. Moreover, such a mild presentation seems to be further substantiated by the lack of histopathological lesions in IDV infected explants, as opposed to the degeneration and necrosis observed in PI3V infected bronchioles in lung explants. Nevertheless, we cannot rule out that the replication of IDV in the alveolar macrophages could cause a dysregulation of immunity, up-regulating pro-inflammatory cytokines that may damage pneumocytes and lead to either primary or secondary pneumonia, similarly to what is observed with selected subtypes of type A influenza viruses (Cline et al., 2017).

One of the most interesting results was that IDV-BOV consistently replicated to higher titers than IDV-SW throughout the tissues and across the whole course of infection, suggesting an overall higher fitness of the bovine strain in sheep. Despite sharing high genetic identity and belonging to the same phylogenetic clade, these two strains differed by 16 amino acids at the consensus level. Since we could not rely on the recently developed reverse-genetics system for IDV (Yu et al., 2019), interpretation of the different replicative phenotype is based both on the VHC and HA data, on the localization of the identified mutations, and in light of the existing literature.

Five amino acid differences between the two strains fall into the HEF protein which is responsible for receptor binding, destroying and membrane fusion activities (Hanika et al., 2005).

Interestingly, IDV-BOV and IDV-SW showed identical tissue binding and hemagglutinating profiles, despite possessing different amino acids at positions 276 and 289, in proximity of the 270-loop, in the receptor binding site of the HEF protein (Song et al., 2016). Nevertheless, we do not exclude that mutations in these positions might affect the receptor preference for specific sialyl linkages and internal glycans, potentially influencing the attachment at the level of the ovine respiratory tract. Evidence in support of this hypothesis can be found in recent work by Liu et al. (2020) who screened IDV strains D/swine/Oklahoma/1334/2011 and D/bovine/Oklahoma/660/2013 with a sialoglycan microarray. Both viruses recorded overlapping interactions with 9-*O*-Acetylated glycans, but each strain displayed fine receptor specificities, that were potentially related to 6 mutations in proximity of the receptor-binding pocket of the HEF protein.

In our strains, no substitution was observed at the level of the catalytic triad of the HEF esterase at positions 57, 356, and 359 (Song et al., 2016), suggesting that differences in the replication were not attributable to a different receptor-destroying ability.

The remaining amino acid differences fall across the PB1, NP, NS1, NS2 proteins, and in the precursor protein P42. Since little information is available on the function of these proteins in IDV viruses, no speculation can be made regarding the possible role of these substitutions. How representative our two viruses are of the swine and bovine influenza D viral populations remains to be determined, as well as how adapted IDV has become to each of these two species. Our phylogeny-based analyses of host association did not reveal a clustering by host of IDV sequences available on GenBank, indicating an extensive viral gene flow between these two species. Nevertheless, this observation could be the result of overrepresentation of sequences from geographically skewed outbreaks. Selection pressure analyses identified sites in which diversifying selection is indeed occurring, in particular at the level of the HEF gene, but whether these positions represent epitopes under immune pressure or functional sites evolving toward host-adaptation we cannot tell.

In light of these observations and considering the limitations intrinsic to *ex vivo* studies, caution is required when translating our results into the complex ecological scenario of IDV. What is clear from our study is that two IDV viruses from different hosts but with closely related genotypes and similar binding preference, differ in their potential to cross the species barrier and infect sheep. Whether such variability is the mere result of using divergent strains, or instead reflects a wide spectrum of phenotypic variability in IDV viral population, we still do not know, but it should be promptly addressed by the scientific community, to better understand the ecology of this disease and monitor its evolution in terms of pathogenicity and host preference. Besides, our study relied on young immature sheep donors that might express slightly different Sia from adults, similarly to what is observed for avian influenza in birds (Pillai and Lee, 2010).

In Italy, for species other than bovine and swine, sampling methods for IDV surveillance and diagnostic activities are currently based on analogies with the bovine model and not on experimental evidence. Given our limited understanding of IDV pathogenesis, surveillance and outbreak investigations might be hampered by improper sampling of animals and tissues. For this reason, we tested the binding profile of IDV-BOV and IDV-SW to the respiratory epithelia of the most commonly farmed mammalian species, to infer both host and tissue preference of IDV in agreement with the 3R principle, and generate the basis for future *in vivo* studies aimed at defining the best sampling methodology.

Unsurprisingly, our findings indicate bovine as the only species in which attachment of both IDVs occurs throughout the entire respiratory apparatus, a result in agreement with the clinical and experimental data describing replication of this virus across the entire respiratory system of bovines (Hause et al., 2017). Interestingly, both IDVs attached more abundantly to the upper rather than the lower respiratory tract, where the staining was sporadic and limited to type-II pneumocytes. This tissue preference is in keeping with *in vivo* studies published so far (Ferguson et al., 2016; Salem et al., 2019), reporting higher viral titers at the level of the nasal turbinates and trachea and a higher frequency of virus detection using nasal swabs, as opposed to lung samples (Collin et al., 2015; Ducatez et al., 2015; Foni et al., 2017).

In the ovine histological sections, binding was limited to the nose and pharynx but to a much lower degree compared to the homologous bovine tissues.

Interestingly, in both small ruminants and in bovine, IDV diffusely attached to the surface of the pharyngeal pseudostratified epithelium. This site is frequently overlooked by practitioners when sampling respiratory viruses but could represent a sensitive target, similarly to what has been recorded for other members of the Orthomyxoviridae family, like human influenza A viruses (Lakdawala et al., 2015).

In the tested ruminants and in horse, IDV showed high affinity for the mucus and submucosal glands of the nose and pharynx. This result is in keeping with IDV’s selective binding affinity for acetylneuraminic acid (Neu5Ac) and *N*-glycolylneuraminic acid (Neu5Gc) carrying *O*-acetyl modifications to position C-9 (Liu et al., 2020), and the Sia composition of saliva and mucins in farmed species. Bovine submaxillary gland mucins are well known for their high content in 9-*O*-acetylated Sia (Lrhorfi et al., 2007) and recent work by Barnard et al. (2020) clearly demonstrated that around 20% of Sia in horse saliva have 9-*O*-acetyl modifications. Nevertheless, mucins can play opposite roles in influenza infection (van Riel et al., 2007). On one hand, mucins act as an innate host defensive barrier, entrapping and inactivating viral particles, but on the other hand, in the absence of full ciliary motility, mucus represents a substrate for virus attachment that can prolong contact with the epithelial surface, hence favoring infection. For this reason, IDV binding to glands and mucus should not be directly translated as receptivity of these tissues.

Surprisingly, IDV in our VHC assay did not bind either to the swine epithelia nor to the submucosal glands and mucus. Although in apparent contrast with a previous study (Song et al., 2016) reporting binding of a baculovirus-expressed IDV HEF protein to the trachea of swine, and with the detection of IDV in this species, our data find justification in the Sia composition of swine epithelia and mucins. Barnard et al. (2020) demonstrated that in the saliva of this species, acetylation is restricted to the C-8 position of Neu5Gc Sia. Moreover, swine upper and lower trachea and lung express Neu5Ac, five–ninefold higher levels than Neu5Gc, but no 9-*O*-acetylated Neu5Ac was detected in these tissues (Sriwilaijaroen et al., 2011). These data indicate that attachment of IDV in this species could be dramatically lower than in other farmed species and strictly dependent on the seemingly limited availability of 9-*O*-acetylated Neu5Gc. Regarding the discrepancy with the attachment observed by Song et al. (2016) we speculate that a superior sensitivity of their technique might have allowed the detection of minimal binding of the HEF protein, or that the presentation of the protein separated from the virion could have allowed unnatural binding to non-9-*O*-acetylated Sia variants in the trachea.

Although our results clearly matched the expected tissue tropism in bovine, we also recorded discrepancies between the profile of attachment and either epidemiological or experimental evidences. For instance, a lack of attachment of IDV in sheep tracheal and lung tissues contrasted with replication of the virus in the corresponding explants, moreover, IDV, although mildly pathogenic, is certainly capable of replicating in pigs, while our VHC system failed to reveal attachment. In light of these observations, we recommend caution when translating VHC data into tissue susceptibility, since a lack of attachment might depend on a number of technical factors that could affect the sensitivity of lectin and virus binding assays, such as the pH of buffers (Zeng and Gabius, 1992), or the use of frozen as opposed to paraffin-embedded sections, determining the availability of mucus on epithelial surfaces (Cohen et al., 2013). For these reasons, we discourage to simply translate negative VHC results into a lack of receptivity *in vivo*, but rather as measure of the probability of attachment.

Summarizing, we can assume ruminants, in particular bovines, are the most receptive species to IDV and the upper respiratory tract is the most likely site to detect IDV. Future *in vivo* studies will elucidate whether the observed high receptivity of the pharynx is associated with high virus replication.

Despite IDV was first isolated in pigs with influenza-like symptoms, several studies have confirmed a low seroprevalence in this species (Foni et al., 2017; Snoeck et al., 2018; Gorin et al., 2019), supporting our observations regarding the lack of binding as an indicator of lower susceptibility of pigs to IDV.

To further compare the binding preference of IDV-BOV and IDV-SW to sialic acids, we investigated the ability of these viruses to agglutinate RBCs of different animal species. The two viruses showed the same agglutinating ability irrespective of the species. Nevertheless, agglutination of erythrocytes is the result of binding to specific sialic acid moieties that do not represent the complexity of receptors distributed in the respiratory epithelia of the homologous species, hence agglutination in one species does not translate into binding to the respiratory tract of the same species (Aich et al., 2011; de Graaf and Fouchier, 2014). Interestingly, HPLC analyses of Sia on erythrocytes from several species (Barnard et al., 2020) shed new light on ours, as well as previously published HA data (Salem et al., 2019). Cow RBCs express Neu5Gc with acetylation in position C-8, hence explaining the lack of agglutination. On the other hand, chicken RBS express 9-*O*-acetylated Neu5Ac supporting IDV agglutination. Surprisingly, IDV agglutinates horse RBCs with Sia acetylated in positions C-8 and C-5, but not C-9, a result that should prompt further research into understanding IDV’s binding preference.

## Conclusion

Our study highlighted for the first time a profound phenotypic variability within a genetically homogenous group of IDV viruses circulating in Italy. Further pathogenesis and cross-species transmission studies will be necessary to investigate the potential for transmissibility of IDV strains and identify the main species involved in spreading the virus. Adopting an *ex vivo* approach for this purpose could represent an ethically and scientifically valid tool to identify diverging phenotypes in need of further *in vivo* characterization.

In general, we advocate for a closer monitoring of the replicative features of this pathogen, beyond molecular and phylogenetic characterization and the implementation of structured passive surveillance based on the systematic investigation of respiratory illness outbreaks in ruminants. In conclusion, determining the real burden of IDV in each of the susceptible species in the field should be prioritized, in order to establish a cost-effective management of this disease.

## Data Availability Statement

The datasets generated for this study can be found in the GenBank: accession numbers from MK965257 to MK965388.

## Ethics Statement

Ethical review and approval was not required for the animal study because according to the national regulation, *ex vivo* studies requiring the sacrifice of animals as donors of tissues are not subjected to the approval of an Ethics Committee. Nevertheless, the Animal Welfare Body of Istituto Zooprofilattico Sperimentale delle Venezie approved the experimental procedures (No. 1 of July 13, 2018) and authorization No. 8B654. NDJA was obtained from the Italian Ministry of Health.

## Author Contributions

FB and IM designed the study. EvM, FB, and AB carried out the culture and infection of explants. EvM conducted virus binding assays, data interpretation, and statistical analysis. SM and ElM gave technical support in performing laboratory experiments. AF and AB conducted and interpreted phylogenetic and molecular analysis. MV contributed to the supervision of histopathological analysis. ES collected the clinical samples for virus isolation and supplied epidemiological information. MB and CC isolated and provided the PI3V and SW-IDV viruses, respectively, and participated to the discussion of data. FB and EvM wrote the manuscript. CT contributed to drafting the discussion. FB contributed to the overall supervision of the study.

## Conflict of Interest

The authors declare that the research was conducted in the absence of any commercial or financial relationships that could be construed as a potential conflict of interest.

## Footnotes

1 https://github.com/vsbuffalo/scythe

2 https://github.com/najoshi/sickle

3 http://broadinstitute.github.io/picard/

4 http://tree.bio.ed.ac.uk/software/figtree/
