# Supplementary material for: Replication of Influenza D Viruses of Bovine and Swine Origin in Ovine Respiratory Explants and Their Attachment to the Respiratory Tract of Bovine, Sheep, Goat, Horse, and Swine
Source: Front Microbiol. 2020 May 25;11:1136. doi: 10.3389/fmicb.2020.01136 (PMC7261881; doi:10.3389/fmicb.2020.01136)
Supplement: Supplementary file 1 [file Data_Sheet_1.docx]

Supplementary Material

| **Gene** | **Statistic** | **observed mean** | **lower 95% CI** | **upper 95% CU** | **null mean** | **lower 95% CI** | **upper 95% CI** | **p-value** |
| --- | --- | --- | --- | --- | --- | --- | --- | --- |
| **HE** | AI | 1.87 | 1.24 | 2.52 | 2.62 | 1.94 | 3.25 | 0.04 |
|  | PS | 14.54 | 13.00 | 16.00 | 15.73 | 14.10 | 16.84 | 0.19 |
|  | MC (bovine) | 10.55 | 8.00 | 16.00 | 7.68 | 5.25 | 12.75 | 0.25 |
|  | MC (swine) | 2.48 | 2.00 | 3.00 | 1.65 | 1.08 | 2.25 | 0.33 |
| **NP** | AI | 0.34 | 0.02 | 0.62 | 1.24 | 0.74 | 1.72 | 0.00 |
|  | PS | 4.80 | 4.00 | 5.00 | 6.60 | 5.67 | 7.00 | 0.02 |
|  | MC (bovine) | 19.00 | 19.00 | 19.00 | 8.33 | 5.18 | 19.00 | 0.04 |
|  | MC (swine) | 2.24 | 2.00 | 4.00 | 1.24 | 1.00 | 2.00 | 0.10 |
| **NS** | AI | 0.76 | 0.32 | 1.25 | 1.35 | 0.94 | 1.77 | 0.01 |
|  | PS | 6.34 | 5.00 | 7.00 | 7.54 | 6.75 | 7.98 | 0.01 |
|  | MC (bovine) | 9.41 | 7.00 | 13.00 | 8.66 | 5.57 | 11.51 | 0.33 |
|  | MC (swine) | 2.18 | 1.00 | 3.00 | 1.27 | 1.00 | 2.00 | 0.06 |
| **P3** | AI | 0.59 | 0.31 | 0.84 | 1.25 | 0.76 | 1.78 | 0.02 |
|  | PS | 5.87 | 5.00 | 6.00 | 6.58 | 5.61 | 7.00 | 0.25 |
|  | MC (bovine) | 10.27 | 8.00 | 12.00 | 8.43 | 4.95 | 12.26 | 0.22 |
|  | MC (swine) | 2.07 | 2.00 | 3.00 | 1.25 | 1.00 | 2.00 | 0.10 |
| **P42** | AI | 0.84 | 0.38 | 1.36 | 1.24 | 0.88 | 1.61 | 0.03 |
|  | PS | 5.77 | 5.00 | 7.00 | 6.65 | 5.88 | 7.00 | 0.19 |
|  | MC (bovine) | 8.32 | 5.00 | 13.00 | 9.25 | 6.61 | 14.14 | 0.64 |
|  | MC (swine) | 1.93 | 1.00 | 3.00 | 1.21 | 1.00 | 2.00 | 0.06 |
| **PB1** | AI | 0.62 | 0.33 | 0.85 | 1.14 | 0.65 | 1.60 | 0.06 |
|  | PS | 4.83 | 4.00 | 5.00 | 6.47 | 5.23 | 7.00 | 0.03 |
|  | MC (bovine) | 9.02 | 9.00 | 9.00 | 5.52 | 3.44 | 11.98 | 0.10 |
|  | MC (swine) | 2.26 | 2.00 | 4.00 | 1.32 | 1.00 | 2.01 | 0.12 |
| **PB2** | AI | 0.42 | 0.11 | 0.74 | 1.15 | 0.66 | 1.62 | 0.01 |
|  | PS | 4.61 | 3.00 | 5.00 | 6.57 | 5.66 | 7.00 | 0.02 |
|  | MC (bovine) | 7.00 | 7.00 | 7.00 | 7.49 | 4.08 | 13.26 | 0.43 |
|  | MC (swine) | 2.49 | 2.00 | 5.00 | 1.24 | 1.00 | 2.00 | 0.11 |

**Supplementary Table 1** Values of Association Index (AI), Parsimony Score (PS) and Monophyletic Clade (MC) obtained using BaTS program from the analyses of the seven genes of all the Influenza D viruses.

| **Gene** | **Positively selected sites** | **P-value (<0.1)** | |
| --- | --- | --- | --- |
|  |  | **MEME** | **FEL** |
| HEF | 233 | 0 |  |
|  | 286 | 0.04 |  |
|  | 456 | 0.04 |  |
|  | 264 | 0.06 |  |
|  | 289 | 0.07 | 0.06 |
|  | 426 | 0.07 |  |
|  | 308 | 0.08 |  |
|  | 563 | 0.08 |  |
|  | 388 | 0.09 | 0.08 |
| NP | 247 | 0.07 |  |
|  | 295 | 0.08 |  |
| NS1 | 122 | 0.07 |  |
| P3 | 477 | 0.05 |  |
| P42 | 364 | 0.06 |  |
|  | 249 | 0.08 |  |
| PB2 | 56 | 0.01 |  |

**Supplementary Table 2**. Amino acid sites of IDV sequences under putative positive selection detected using different analytical models.


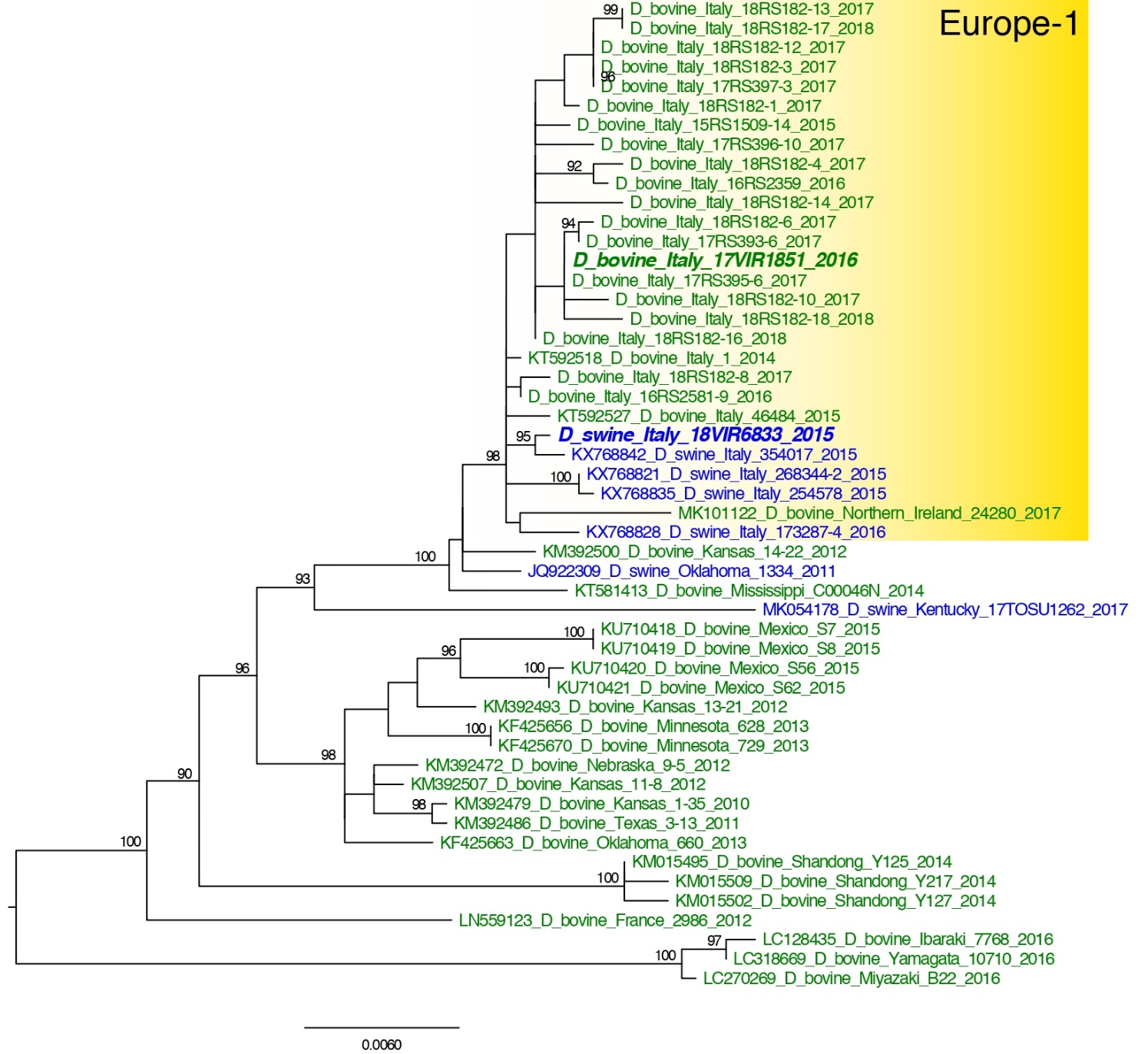


**Supplementary Figure 1.** Maximum likelihood phylogenetic tree of the NP gene segment of Influenza D viruses. Viruses are coloured according to the host species: blue for swine, green for bovine. The two strains characterized in this study are in bold. Europe-1 cluster, including all the Italian viruses, is highlighted in yellow. The numbers at the nodes represent ultrafast bootstrap values (>90%).


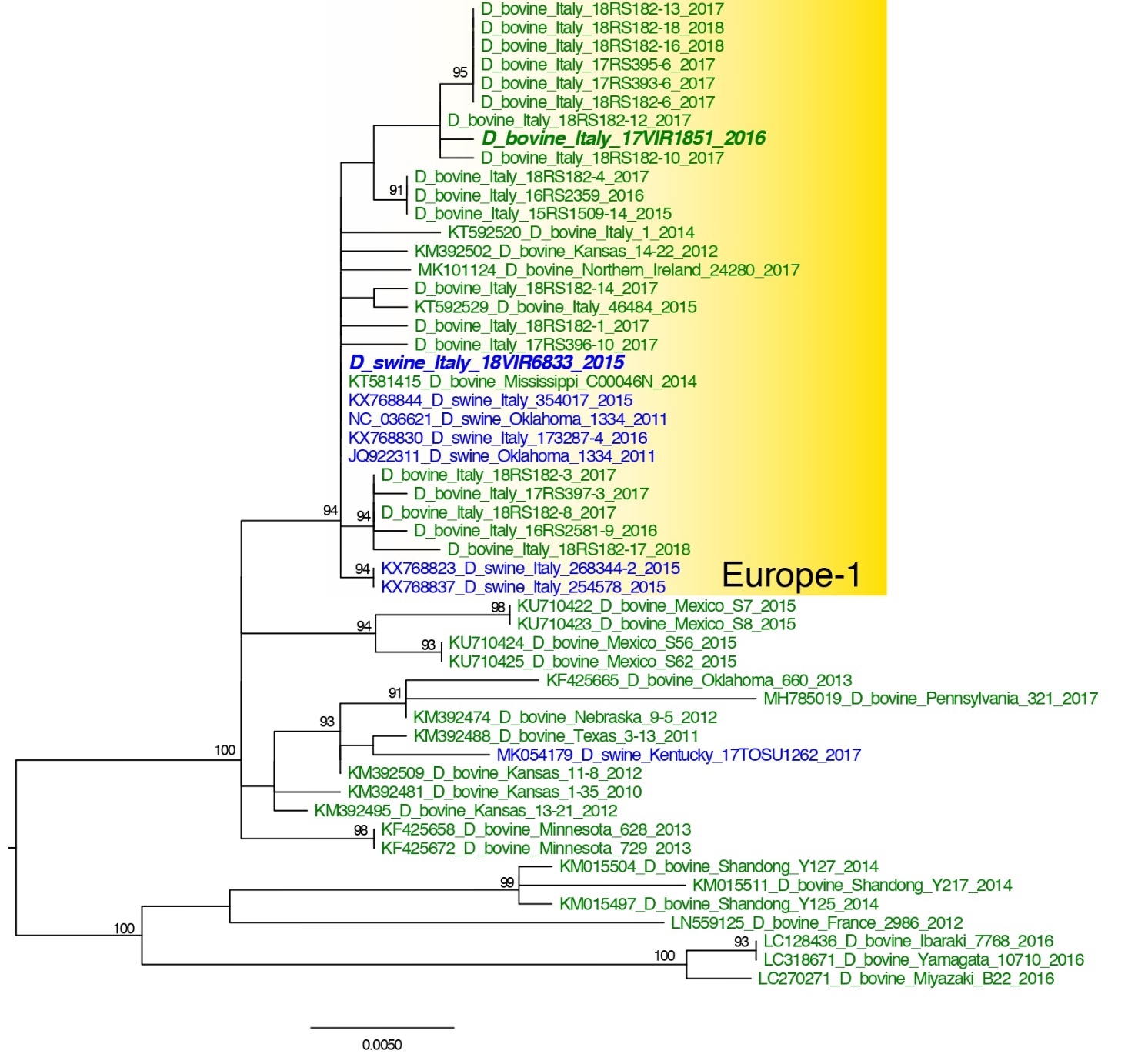


**Supplementary Figure 2.** Maximum likelihood phylogenetic tree of the NS gene segment of Influenza D viruses. Viruses are coloured according to the host species: blue for swine, green for bovine. The two strains characterized in this study are in bold. Europe-1 cluster, including all the Italian viruses, is highlighted in yellow. The numbers at the nodes represent ultrafast bootstrap values (>90%).


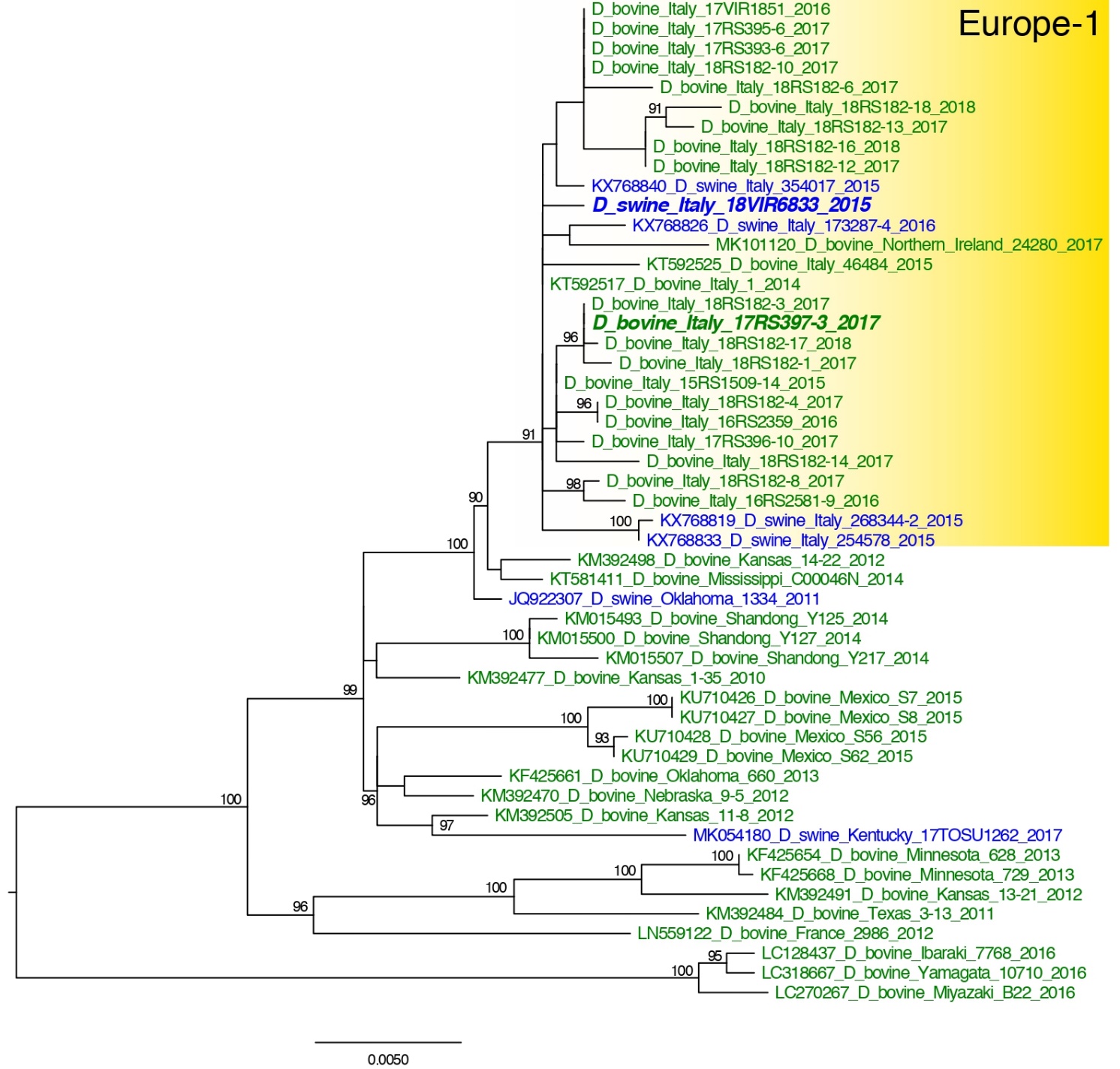


**Supplementary Figure 3.** Maximum likelihood phylogenetic tree of the P3 gene segment of Influenza D viruses. Viruses are coloured according to the host species: blue for swine, green for bovine. The two strains characterized in this study are in bold. Europe-1 cluster, including all the Italian viruses, is highlighted in yellow. The numbers at the nodes represent ultrafast bootstrap values (>90%).

**
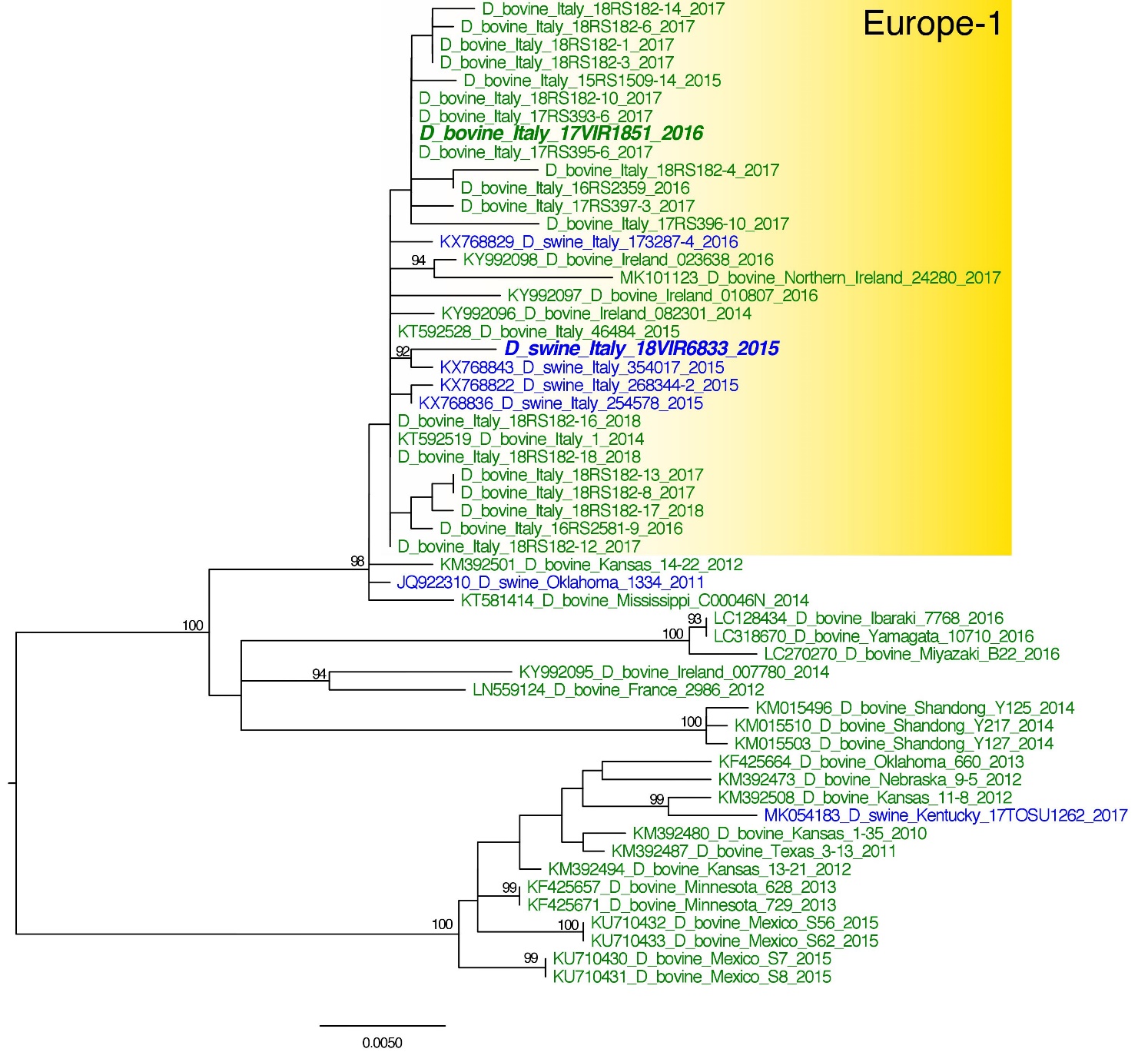
Supplementary Figure 4.** Maximum likelihood phylogenetic tree of the P42 gene segment of Influenza D viruses. Viruses are coloured according to the host species: blue for swine, green for bovine. The two strains characterized in this study are in bold. Europe-1 cluster, including all the Italian viruses, is highlighted in yellow. The numbers at the nodes represent ultrafast bootstrap values (>90%).

**
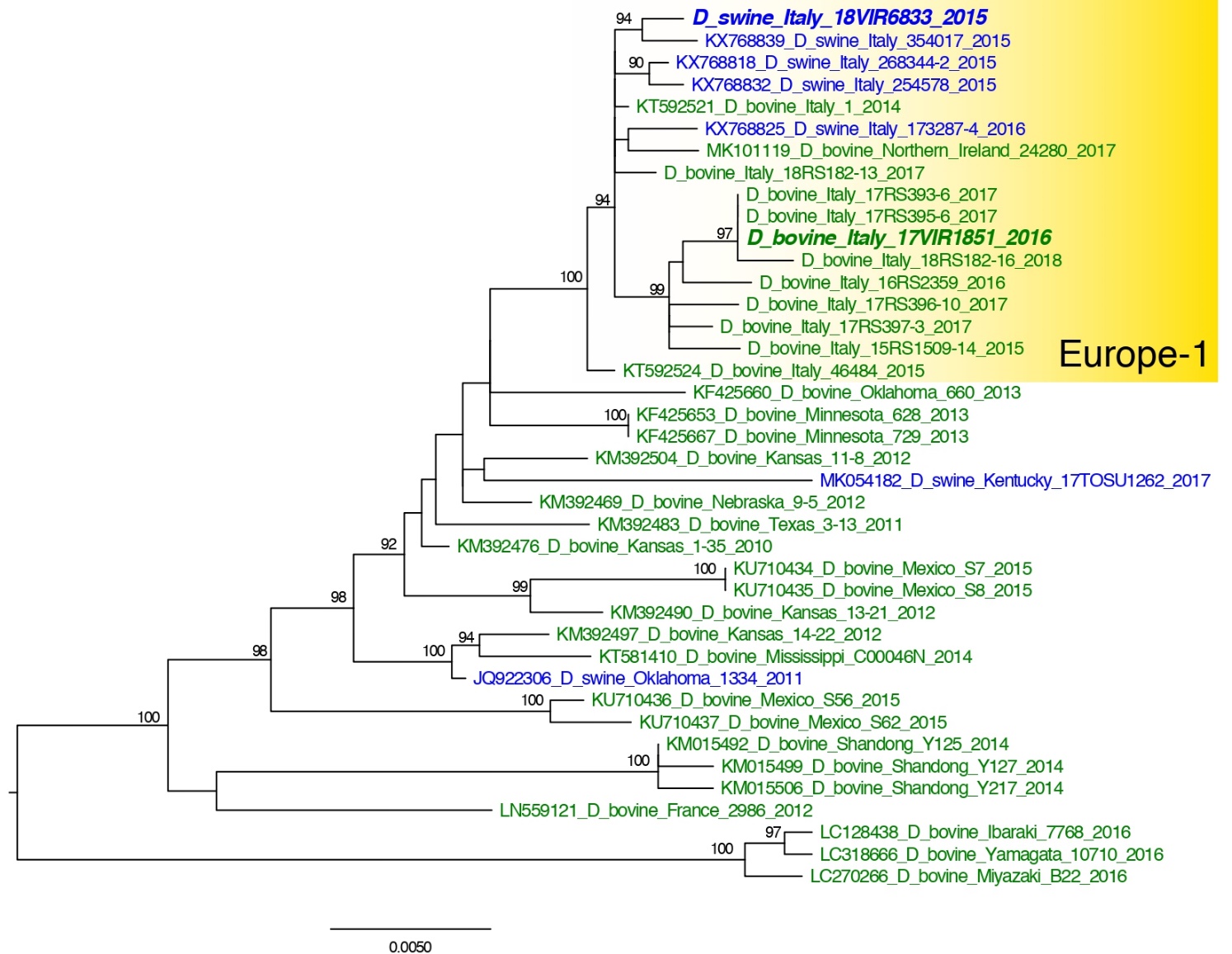
Supplementary Figure 5.** Maximum likelihood phylogenetic tree of the PB1 gene segment of Influenza D viruses. Viruses are coloured according to the host species: blue for swine, green for bovine. The two strains characterized in this study are in bold. Europe-1 cluster, including all the Italian viruses, is highlighted in yellow. The numbers at the nodes represent ultrafast bootstrap values (>90%).

**
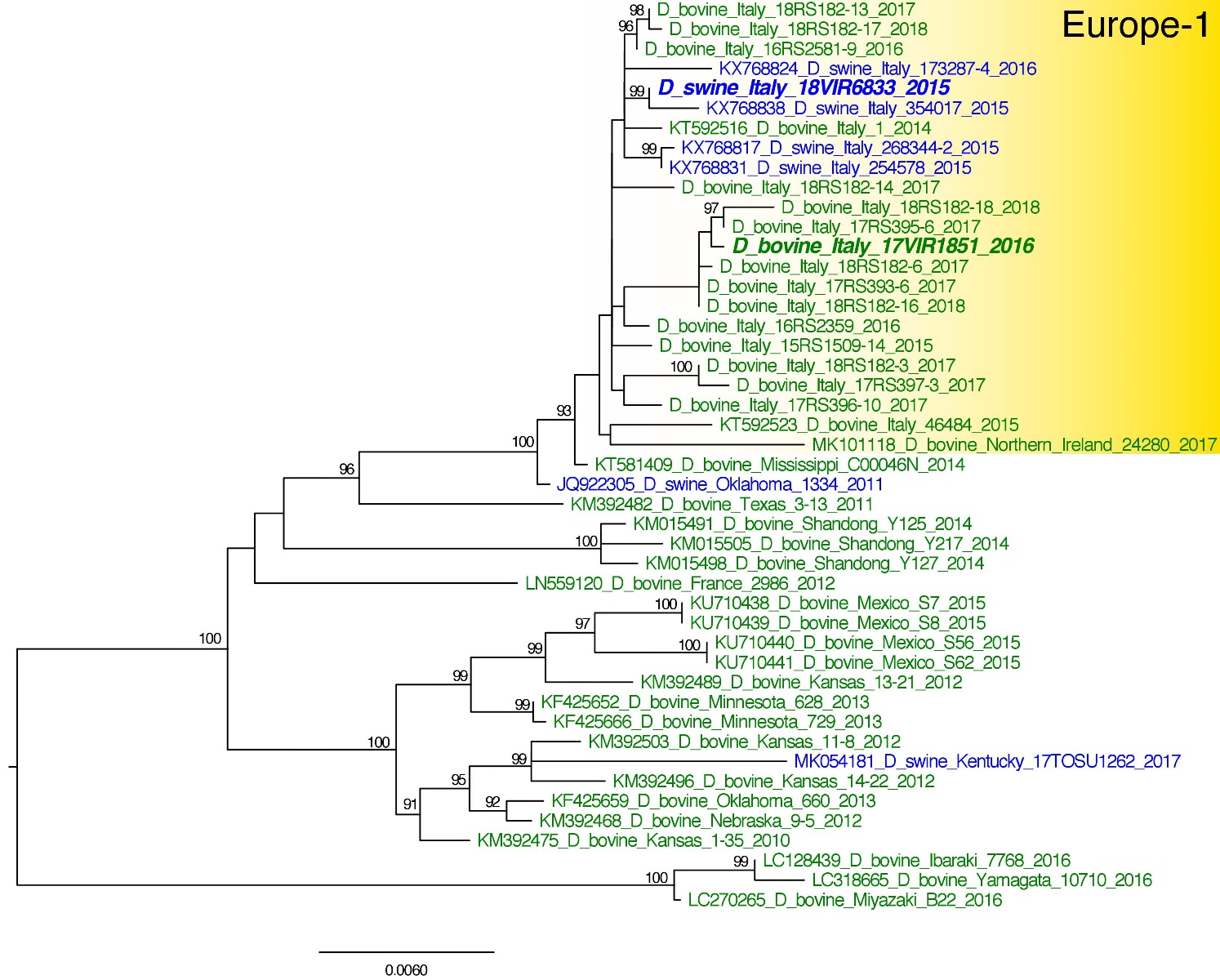
Supplementary Figure 6.** Maximum likelihood phylogenetic tree of the PB2 gene segment of Influenza D viruses. Viruses are coloured according to the host species: blue for swine, green for bovine. The two strains characterized in this study are in bold. Europe-1 cluster, including all the Italian viruses, is highlighted in yellow. The numbers at the nodes represent ultrafast bootstrap values (>90%).
